# Vision and Vision-Related Measures in Progressive Multiple Sclerosis

**DOI:** 10.3389/fneur.2019.00455

**Published:** 2019-05-03

**Authors:** Yael Backner, Panayiota Petrou, Haya Glick-Shames, Noa Raz, Hanna Zimmermann, Rebecca Jost, Michael Scheel, Friedemann Paul, Dimitrios Karussis, Netta Levin

**Affiliations:** ^1^fMRI Unit, Neurology Department, Hadassah-Hebrew University Medical Center, Jerusalem, Israel; ^2^Neurology Department, The Multiple Sclerosis Center, Hadassah-Hebrew University Medical Center, Jerusalem, Israel; ^3^NeuroCure Clinical Research Center, Charité-Universitätsmedizin Berlin, Corporate Member of Freie Universität Berlin, Humboldt-Universität zu Berlin, and Berlin Institute of Health, Berlin, Germany; ^4^Experimental and Clinical Research Center, Max Delbrueck Center for Molecular Medicine and Charité-Universitätsmedizin Berlin, Berlin, Germany

**Keywords:** optic neuritis, motion perception, OCT, VEP, fellow eye, multiple sclerosis

## Abstract

**Background:** Over the last few years there has been growing interest in use of visual measures as useful tools for multiple sclerosis (MS) prognosis and tracking. Optic neuritis (ON) being a prevalent and often-presenting symptom of the disease, as well as the high occurrence rate of posterior visual system damage independent of ON (optic radiation lesions), make the visual system a prime candidate for such endeavors. However, while the visual system makes for a convenient model in early stages of MS, processes which may be true in those stages may drastically change as the disease progresses, due to accumulated disease load. Here, we examine whether vision-related tools reflect demyelinative and axonal damage of the visual pathways and may be used for assessment in the clinical setup in progressive multiple sclerosis (MS) patients, in whom disease load may alter the early stage picture.

**Methods:** Forty-eight progressive MS patients, with and without prior optic neuritis (ON), underwent a battery of behavioral tests, visual evoked potential (VEP) tests, optical coherence tomography (OCT), and structural MRI scans, at two time-points. Data were analyzed for stability between visits and for correlation between behavioral and electrophysiological data.

**Results:** All measures were stable between visits. Significant differences were found in all measures between the affected and fellow eyes of ON patients and in VEP latencies between the affected and non-ON eyes. Motion perception differentially correlated with latencies of both ON eyes and with the non-ON eyes. Retinal nerve fiber layer thickness correlated with the latencies of non-ON eyes but not of either ON eye. No difference in lesion load was found between the ON and non-ON patients.

**Conclusions:** ON still leaves its mark in the patient's visual system over time, with all visual measures of the affected eyes notably reduced compared to fellow eyes. Motion perception, reflecting myelination level along the visual pathway, shows its usefulness also in progressive MS. In the non-ON eyes, axonal loss appears to explain prolonged latencies, unlike in ON eyes, where demyelination appears to be the main mechanism. Lastly, the visual measures assessed herein are applicable as valid assessment tools in therapeutic studies.

## Introduction

Over the last few years there has been growing interest in the use of visual measures as useful tools for multiple sclerosis (MS) prognosis and tracking. Optic neuritis (ON) being such a prevalent and often-presenting symptom of the disease, as well as the high occurrence rate of posterior visual system damage independent of ON (optic radiation lesions), make the visual system a prime candidate for such endeavors (1, 2). Current studies make use of a wide variety of visual measures, from behavioral tests such as low-contrast letter acuity (LCLA) (3) and motion perception (4), through imaging methods such as optical coherence tomography (OCT) (5–8) and magnetic resonance imaging (MRI) (9, 10), to the electrophysiological visual evoked potential (VEP) test (11).

However, most previous studies dealing with visual tools in MS focused on acute cases of ON and early-stage MS, looking at different aspects of myelin and axonal damage in the visual system and its predictive value for disease prognosis. While the visual system, as well-defined as it is, makes for a convenient model in the early stages of MS, processes which may be true in those early stages may drastically change as the disease progresses, due to accumulated disease load.

In the current study, we seek to examine whether previously established observations regarding vision and vision-related measures in clinically isolated acute ON patients hold true in progressive MS patients in whom disease progression may alter the picture.

## Methods

### Subjects

Forty-eight progressive MS patients, designated MS-ALL, were enrolled in a longitudinal mesenchymal stem cell therapy study (NCT02166021), conducted at the Hadassah-Hebrew University Medical Center from January 2015 to June 2018, comprising nine visits over the duration of 1 year. Six visits included visual assessments. The scope of this study is the screening and baseline assessments, prior to therapeutic intervention, scheduled roughly 2 months apart. Inclusion criteria for the full study were the 2010 revised McDonald criteria for MS (12), age 25–64, disease duration of at least 3 years, progressive forms of MS, EDSS score of 3.5–6.5, and failure to respond to the currently-available registered treatments. Exclusion criteria were treatment with cytotoxic or immunomodulatory medications in the 3 months prior to inclusion, significant diseases that may risk the patient or interfere with results, active infections, severe cognitive decline, as tested by the Brief International Cognitive Assessment for MS (BICAMS) (13), and previous cellular treatment of any kind.

For analysis purposes, the MS-ALL group was divided into two subgroups, based on reported prior history of ON, designated MS-ON and MS-nON. In the MS-ON subgroup, affected (AE) and fellow (FE) eyes were defined. Following initial between-visit stability analysis, bilateral ON patients were removed from subgroup analyses, since our interest lies in the difference between ON AEs and FEs and in these subjects effects cannot be separated.

This study was approved by the Hadassah-Hebrew University Medical Center Ethics Committee. All participants gave written informed consent.

### Data Acquisition and Analysis

All behavioral, electrophysiological, and imaging measures were taken at two consecutive time points, ~2 months apart. All tests were performed with best-corrected visual acuity for all subjects.

#### Visual Acuity (VA) and Low-Contrast Letter Acuity (LCLA)

Visual acuity (VA) and low-contrast letter acuity (LCLA) were measured using a multi-contrast Sloan letter chart at 100 and 2.5%, respectively. VA results were converted to decimal scale. LCLA results were converted to a scale of 0–60 using bins of 5 (5 points assigned to each line that could be fully determined).

#### Color Perception

Color perception was assessed using pseudochromatic plates 5–10 of the Hardy Rand and Rittler (HRR) color test with a score of 1 given for full recognition of shapes and 0 for partial or no recognition, in any given plate.

#### Motion Perception

Motion perception was assessed using object-from-motion (OFM) and number-from-motion (NFM) extraction tasks, based on the opposing movement of dot arrays outside and inside the formed object/number. The camouflaged object/number cannot be discerned when the dots are stationary [these tests have been previously described (14)]. Briefly, stimuli were presented at seven speeds, the slowest being most difficult. Scoring for each stimulus correctly identified was according to the relative weight given to each speed (e.g., 7 points for level 1, 6 for level 2, etc.). Maximum score was 140.

#### Visual Evoked Potentials (VEP)

Visual evoked potentials (VEP) were recorded by a trained technician on a Bravo VEP device (Nicolet Biomedical) using standard full-field pattern-reversal VEP parameters. Lateral electrodes were placed at O1 and O2 with a reference electrode at Fz and ground electrode at the vertex. Abnormal values were defined as above 114 ms. VEP P100 latencies and amplitudes were extracted. At least two repetitions were recorded for each eye, the reported values being an average of the two recordings.

#### Peripapillary Retinal Nerve Fiber Layer (pRNFL) Thickness and Macular Volume

Peripapillary retinal nerve fiber layer (pRNFL) thickness and macular volume were recorded by trained technicians using spectral-domain OCT (Spectralis, Heidelberg Engineering) with automatic real time (ART) function for image averaging. pRNFL was derived from standard ring scans around the optic nerve head. Macular volume was derived from custom macula scans (30° × 25°, 61 B-scans, ART: 13 frames). All scans underwent quality control (15). Automatic segmentation results were checked for errors and corrected if necessary by an experienced observer blind to the subjects' condition.

#### Lesion Load

Lesion load was measured on T2-FLAIR imaging scans, using the lesion segmentation toolbox (LST) (16) of the statistical parametric mapping (SPM) software for automatic lesion detection, and was then manually corrected. Brain tissue volume, normalized for subject head size, was estimated using SIENAX (17), part of FSL (18).

#### Regional Tissue Volume Assessment

Regional tissue volume assessment was performed using the region-based morphometry (RBM) module of the Computational Analysis Toolbox (CAT12, Jena University Hospital), an extension of SPM12 (Wellcome Department of Cognitive Neurology). Data were prepared using default preprocessing parameters, including brain segmentation (into gray matter, white matter, and cerebral spinal fluid), and adding surface and thickness estimation for region-of-interest (ROI) analysis. Estimated ROI volume values were extracted for each subject, using the LONI probabilistic brain atlas (LPBA40) (19) and the results for all vision-related regions were compared between the two subgroups (Supplementary Table 1).

### Statistical Analysis

Mean values of all measures for MS-ALL were compared for the two visits through paired 2-tailed *t*-tests to assess stability of methods over a short duration. Mean values of all measures were compared between MS-ON affected eyes (AE) and fellow eyes (FE) through paired 2-tailed *t*-tests to assess the persistence of ON-caused damage. Mean values of all measures were compared between MS-nON eyes and the MS-ON AEs and FEs through unpaired 2-tailed *t*-tests. Pearson correlation coefficients were used to examine the associations between the VEP test and other measures of the visual system.

Due to inter-eye latency correlations, linear mixed effects models were built to test the relationship between the various measures, group and VEP latency, with patient ID as a random variable. Separate models were built between families of test types (static visual tests—VA, LCLA; dynamic visual tests—NFM, OFM; OCT measurements-macular volume, pRNFL thickness) with VEP latency as dependent variable. All significant parameters from the above models were fed to a fourth model to explore interactions between eye subgroup (AE, FE, non-averaged MS-nON eyes) and measures. Further models were built for specific measures which have shown significant interactions by group to test the interaction's effect on the measure.

Significance for all tests and correlations was assessed at *p* < 0.05 level. Multiple comparisons correction was for familywise error rate (FWE) using the Holm method. All calculations were done using R-version 3.3.2 (R Foundation for Statistical Computing, Vienna, Austria).

## Results

### Subject Characteristics

MS-ALL group included 48 patients with progressive forms of MS (18 progressive with relapses, 21 secondary-progressive, 9 primary-progressive, 20 females, average age 47.5 ± 9.5 years [mean ± SD]). Last treatments before inclusion included fingolimod (21 patients), dimethyl fumarate (10), interferon β1a (4), glatiramer acetate (4), natalizumab (3), teriflunomide (2), rituximab (2), interferon β1b (1), and azathioprine (1). MS-ON subgroup included 21 patients (8 females, 11 with right affected eye, 8 with left affected eye, 2 with bilateral ON, mean time from episode 16.8 ± 6.5 years). MS-nON subgroup included 27 patients (12 females). Visual assessments were made at two time points, 66.1 ± 10.4 days apart. No significant differences were found in EDSS, age, or in the BICAMS visuospatial memory test (BVMTr) between the two subgroups (two-tailed *t*-test; *p* = 0.95, *p* = 0.39, and *p* = 0.13, respectively). See Table 1 for cohort characteristics.

**Table 1:** Study cohort characteristics.

	**MS-ALL**	**MS-ON**	**MS-nON**
Subjects (n)	48	21	27
Sex (F/M)	20/28	8/13	12/15
Age (years ± SD; range)	47.5 ± 9.5 (26–67)	46.14 ± 8.53 (26–63)	48.56 ± 10.18 (30–67)
MS Form (PwR/SPMS/PPMS)	18/21/9	8/13/0	10/8/9
EDSS at start (median; range)	5.75 (3.5–6.5)	5.5 (4–6.5)	6 (3.5–6.5)
BVMTr (average ± SD; range)	−0.03 ± 1.37 (−2.87 to 2.15)	0.31 ± 1.45 (−2.87 to 2.15)	−0.29 ± 1.28 (−2.82 to 1.91)
Bilateral ON	2	2	N/A
AE (R/L)	N/A	11/8	N/A

*MS-ALL, full cohort; MS-ON, subjects with prior optic neuritis (ON); MS-nON, subjects without prior optic neuritis; PwR, progressive multiple sclerosis with relapses; SPMS, secondary-progressive multiple sclerosis; PPMS, primary-progressive multiple sclerosis; EDSS, expanded disability status scale; BVMTr, Brief visuospatial memory test-revised; AE, affected eye; R, right; L, left*.

### Between-Visit Stability

All vision-related measures tested maintained consistency over the two consecutive time-points in which measurements were taken. *T*-test paired comparison was performed and no significant differences were detected between visits in any of the tested measures. Since no significant differences were detected, further analyses are shown only for the first visit's data.

### Subgroup Visual Measures Comparison

To simplify analyses, differences between all measurements taken for the right and left eyes of MS-nON patients were tested. As no significant differences were found between eyes, the two eyes of each MS-nON patient were averaged into a single value for further comparisons against MS-ON AEs and FEs.

**Figure 1:**
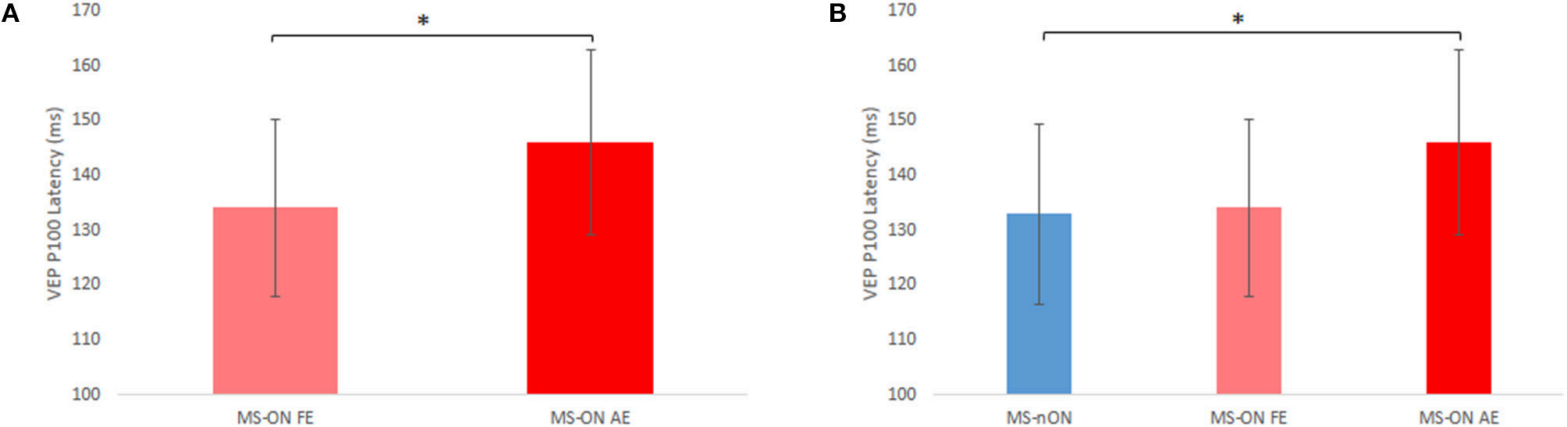
VEP P100 latency differences between subgroups. **(A)** Paired *t*-test comparison of MS-ON fellow and affected eyes (*n* = 16) **(B)** Non-paired *t*-test comparison of MS-nON eyes (*n* = 27), MS-ON fellow eyes (*n* = 19), and MS-ON affected eyes (*n* = 16). Error bars indicate SD. *Represents significance (*p* < 0.02).

VEP latencies of all three eye subgroups were examined. The latencies of 3 MS-ON AEs were excluded due to unsatisfactory recording quality. With the exception of 2 MS-nON patients, all patients in the MS-ALL group had abnormal latency values in both eyes. However, significant differences were found between MS-ON AEs and MS-ON FEs (paired *t*-test, *N* = 16; *p* = 0.014, Figure 1A) as well as between MS-ON AEs and MS-nON eyes (non-paired *t*-test, N_MS−ON−AE_ = 16; N_MS−nON_ = 27; *p* = 0.018, Figure 1B). No significant differences were found between MS-ON FEs and MS-nON eyes (non-paired *t*-test, N_MS−ON−FE_ = 19; N_MS−nON_ = 27; *p* = 0.81, Figure 1B).

All other measures from the visual assessments also underwent paired *t*-test comparisons between MS-ON AEs and FEs and non-paired *t*-test comparisons between MS-nON eyes and MS-ON AEs or FEs. In the AEs vs. FEs comparisons, significant differences were found in most measures (VA, LCLA, OFM/NFM, macular volume, pRNFL thickness, *p* < 0.03 for all measures), confirming that the damage caused to the visual pathways can still be discerned years following the acute episode. The only exception was the HRR color test, though it was close to significance (*p* = 0.057). In the MS-nON eyes vs. AEs or FEs comparisons, on the other hand, no significant differences were found in any of the measurements, though the differences between MS-nON eyes and AEs showed a trend of worse scores in the AEs.

### Subgroup MRI Measures Comparison

To verify the similarity in disease load, brain volume, and lesion count and load were compared between subgroups. No significant differences were found in any of the measures. Estimations of regional vision-related tissue volumes were also compared between subgroups. No significant differences were found (see Supplementary Table 1 for details regarding the selected ROIs and results).

### Motion Perception and VEP Latencies

**Figure 2:**
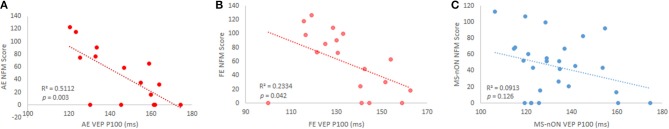
VEP P100 latency and NFM score correlations in subgroups. **(A)** MS-ON AE (*p* = 0.003); **(B)** MS-ON FE (*p* = 0.042); **(C)** MS-nON (*p* = 0.13).

Previous observations made by our group showed that dynamic visual functions reflect myelination levels of the visual pathway, as measured by VEP latencies (20). To examine whether these observations hold true for patients even years after the acute episode, the results of our motion perception tests (OFM/NFM) were correlated to the VEP latencies of all eyes. Significant inverse correlations were found between both motion perception tests and the latencies of the AEs (*N* = 15; OFM: *r* = −0.701, *p* = 0.004; MFM: *r* = −0.715, *p* = 0.003, Figure 2A) as well as between the NFM test and the FE latency (*N* = 18, *r* = −0.48, *p* = 0.042, Figure 2B), though this latter result did not survive multiple comparison correction. Lack of correlation between MS-ON FEs and OFM scores appear to stem from a single outlier (when removed, *r* = 0.65, *p* = 0.005). No similar correlations were observed in the MS-nON group (Figure 2C).

**Figure 3:**
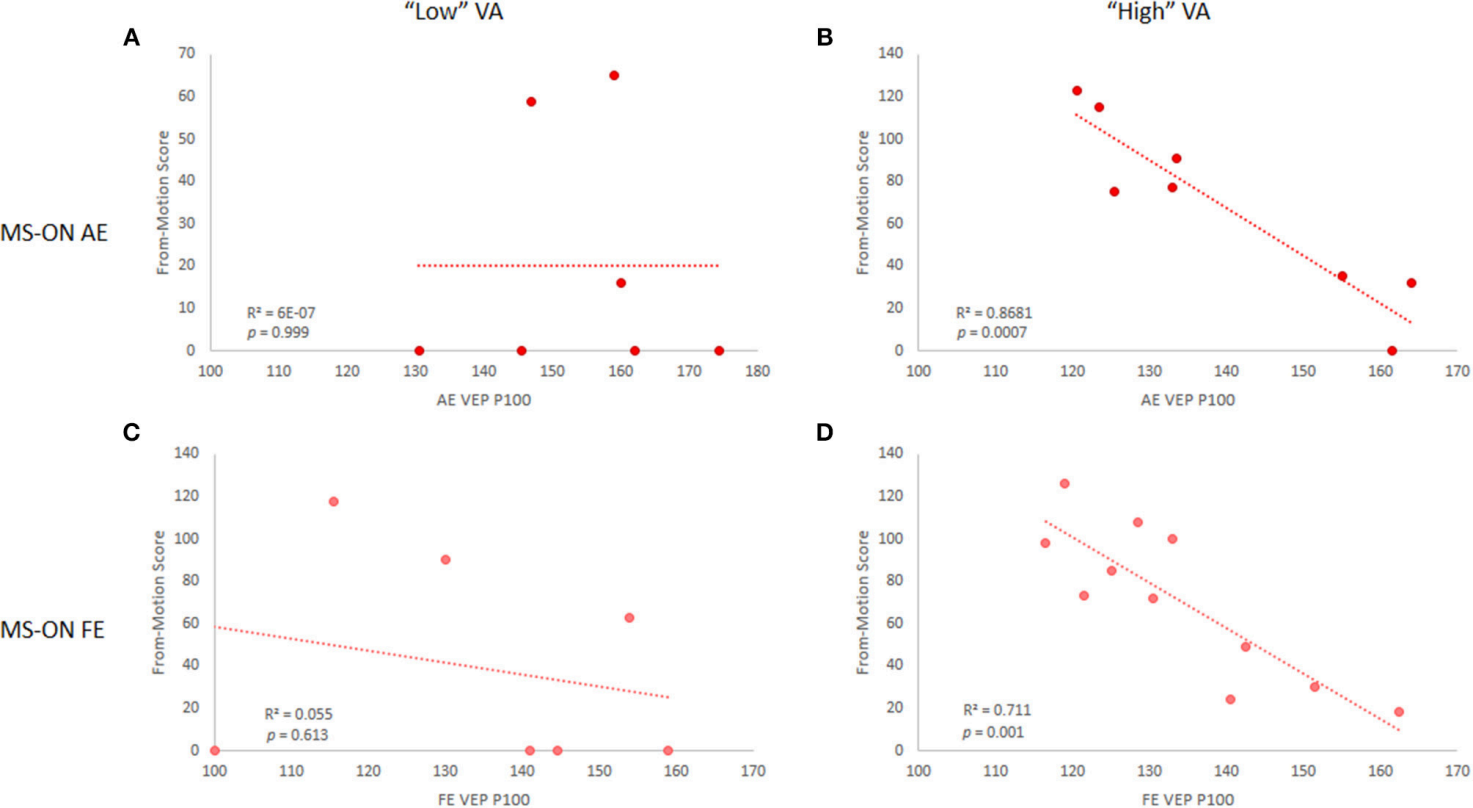
VEP P100 latency and NFM score correlations in MS-ON eyes for “low” and “high” VA. Correlations are shown for MS-ON AE with “low” **(A)** and “high” **(B)** VA (*p* = 0.999 and *p* = 0.0007, respectively) and for MS-ON FE with “low” **(C)** and “high” **(D)** VA (*p* = 0.613 and *p* = 0.001, respectively). Results shown for NFM test only, but similar results are found for the OFM test.

Since some of our cohort exhibited below-normal VA (< 0.8, no significant differences between MS-nON eyes and either MS-ON AEs or FEs), to test whether the results were VA-dependent, subjects in each eye subgroup were divided into “high” (≥0.8) and “low” (<0.8) VA based on normal range definitions (21) and correlations were reanalyzed accordingly. Results revealed that the correlations were driven by the “high” VA group in both MS-ON AEs (*n* = 8; OFM: *r* = 0.84, *p* = 0.01; MFM: *r* = 0.93, *p* = 0.0007, Figures 3A,B) and FEs (*n* = 11; OFM: trend only, *r* = 0.54, *p* = 0.085; NFM: *r* = 0.84, *p* = 0.001, Figures 3C,D). Similar division of subjects in the MS-nON group did not yield the same results, with no correlation found in the “high” VA group (not shown).

To examine whether the motion perception tests may also be affected by axonal damage, test results were also correlated to the pRNFL thickness and macular volume. No significant correlations were found.

### pRNFL Thickness and VEP Latencies

**Figure 4:**
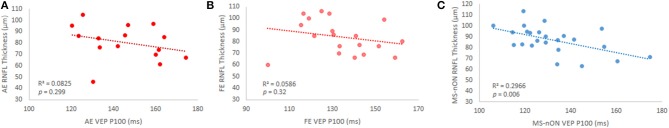
VEP P100 latency and pRNFL thickness correlations in subgroups. **(A)** MS-ON AE (*p* = 0.3); **(B)** MS-ON FE (*p* = 0.32); **(C)** MS-nON (*p* = 0.006).

Whereas, the MS-ON cohort did not show any correlation of the VEP latencies with the pRNFL thickness in either eye (Figures 4A,B), such correlation was found for the MS-nON latencies (*r* = −0.54, *p* = 0.006, Figure 4C), suggesting a relationship between axonal damage and delay of signal conduction in MS-nON patients, that is different from the relationship observed in the MS-ON subgroup.

### Mixed Effects Model

The mixed effects models showed that none of the static visual tests were independently correlated with VEP latencies, whereas NFM and pRNFL thickness both were significantly inversely correlated to the latencies. A multivariate unified model with eye subgroup (AE, FE, non-averaged MS-nON eyes), NFM, and pRNFL thickness showed no significant difference in latency between FEs and MS-nON eyes and that NFM was significantly correlated with shorter latencies, whereas pRNFL thickness was not (Table 2A). Since added interaction terms showed significant interaction of NFM and pRNFL thickness with eye group, separate models were built, correlating latencies with these measures by eye group. This revealed that while NFM was inversely correlated with the latencies in all subgroups, an escalating effect exists, with significance rising from MS-nON, to FE, to AE (Table 2B). pRNFL thickness showed significant effect in the MS-nON subgroup only (Table 2C).

**Table 2:** Mixed effects model results.

**Parameter**	**Estimate [95% CI]**	***p*-value**
**(A) NFM and pRNFL–all subgroups**		
Subgroup FE	1.6 [−7.7, 10.8]	0.734
Subgroup AE	11.2 [1.5, 20.8]	0.024^*^
NFM	−0.2 [−0.3, −0.1]	0.000^*^
pRNFL thickness	−0.2 [−0.5, 0.1]	0.140
**(B) NFM by subgroup**		
Subgroup FE	9.56 [−3.5, 22.6]	0.146
Subgroup AE	23.31 [11.3, 35.3]	0.000^*^
Subgroup MS-nON:NFM	−0.12 [−0.2, 0]	0.025^*^
Subgroup FE:NFM	−0.25 [−0.4, −0.1]	0.001^*^
Subgroup AE:NFM	−0.34 [−0.5, −0.2]	0.000^*^
**(C) pRNFL by subgroup**		
Subgroup FE	−17.4 [−69.6, 34.8]	0.504
Subgroup AE	−1.8 [−50.2, 46.6]	0.940
Subgroup MS-nON:pRNFL thickness	−0.5 [−0.9, −0.1]	0.012^*^
Subgroup FE: pRNFL thickness	−0.3 [−0.8, 0.2]	0.203
Subgroup AE: pRNFL thickness	−0.4 [−0.8, 0.1]	0.117

*FE, fellow eye; AE, affected eye; MS-nON, subjects without prior optic neuritis; NFM, number-from-motion; pRNFL, peripapillary retinal nerve fiber layer*.

* *Represents significance*.

## Discussion

Here, we have explored the visual system of progressive MS patients and the effects of ON years after the acute episode and found that despite the passage of time, previous ON episodes still leave their mark in the patient's visual system. This mark is characterized by persistently prolonged VEP latencies in the AEs of patients who have experienced prior ON in comparison to both their FEs and the eyes of patients who have not had ON. Additionally, even years after the acute episode, all visual measures of the AE are notably reduced compared to the FE. Motion perception, as a tool reflecting myelination levels along the visual pathway, still showed its usefulness in progressive MS. In the MS-nON eyes, axonal loss, as reflected by pRNFL thinning, appears to explain the prolongation of conduction velocities, unlike in MS-ON eyes, where demyelination appears to be the main mechanism involved. Lastly, we have shown that the visual measures evaluated in this study are replicable over short durations, attesting their applicability as valid assessment tools in therapeutic studies.

### Residual Deficits in ON Affected Eyes Years After the Episode

Previous studies, including the comprehensive North American ON treatment trial (ONTT), have reported that most patients suffering typical ON reach good visual recovery within a year of onset (22), though persistent residual deficits may still remain, encompassing the entire range of visual functions, including VA and contrast sensitivity, color vision, stereopsis, and VEPs (11). It has been suggested that incomplete recovery is the result of persistent demyelination (20). Our results are in line with those reporting residual deficits, showing them in almost all of the tested functions, and further noting that when comparing AE and FE in progressive MS patients, even in the presence of accumulated disease load, it is still possible to discern the AE from the FE. Furthermore, though there were no significant differences found between the various visual measures of the MS-nON eyes and the AEs, the general trend showed better function of the MS-nON eyes compared to the AEs. Considering the substantially unequal sizes of the subgroups, this non-significance might be explained by the reduced power of the non-paired compared to paired *t*-test.

### Motion Perception as Myelination Marker

In Raz et al. (20), our group claimed that motion perception, as shown by the OFM test scores, reflects myelination levels of the optic nerve (20). The cohort in that study included mostly CIS-ON patients and some early-stage MS patients. Assessment was done over the first year following the acute episode. Herein we show that this holds true in progressive patients who underwent assessment many years following the acute episode. It is known that the VEP latencies of ON AEs commonly remain prolonged (23), reflecting incomplete remyelination (20). In our current work we further emphasize the persistent connection between conduction velocities and motion perception, showing the applicability of the OFM/NFM tool even in progressive patients. Though mixed effects models found correlation between all eye subgroup latencies and NFM scores, a significant escalation exists in correlation significance, suggesting subtle demyelination as a global effect of the disease with greater effects in lesioned sites. It should, however, be noted that the use of the motion tools necessitates normal VA in subjects taking the test and so might be limited in low VA subjects.

Contrary to the AE behavior in this cohort, which confirmed our previous work, the FE behavior was decidedly different. Briefly, in our previous early-stage cohort the relative prolongation of the FE's VEP latency did not correlate with the ability to perform motion perception tests. It was posited that the observed prolongations were caused by two separate pathophysiological mechanisms. Whereas, in the AE prolongation was derived from demyelination, in the FE it was suggested that this was caused by compensatory cortical-level mechanisms (24, 25). In the current cohort, however, prolongation of the FE's VEP latency did correlate with the motion perception tests, suggesting a demyelinative effect rather than cortical adaptive mechanism. Thus, whatever cortical mechanisms may be involved in the early stages, here it is possible that they have succumbed to the accumulation of disease. It has been previously shown in other neurodegenerative diseases that compensatory mechanisms cannot withstand the load of disease indefinitely and are more typical in the early stages of disease (26).

And yet, how can demyelination of the FE be explained in the absence of reported acute ON episode in that eye? The difference in motion perception behavior between the MS-nON eyes and FEs despite the similar disease stages and lesion load of the two groups, points to a process unique to the FE. A possible explanation, raised by Alshowaeir et al. in a recently published study, is that of inflammation spillover from the AE (27). In other words, demyelination of one optic nerve can affect the fellow optic nerve. This proposed mechanism, however, needs to be studied further. Notwithstanding, this suggests that the use of FEs as controls for AEs in MS studies should be approached more cautiously.

### VEP Latency Prolongation Mechanisms

Accelerated pRNFL thinning (beyond the normal aging processes) is known to occur in MS patients, even without prior reported ON (7). The two suggested explanations are the independent axonal loss component of the disease which can occur also in the retina, and retro-chiasmal damage as consequence of optic radiation lesions, a common finding in the disease (28, 29). The pRNFL thickness in such cohorts was found to correlate with both multifocal and full-field VEP latencies (30, 31). In our cohort, we saw prolonged VEP latencies in all eye subgroup, but the latencies of MS-nON eyes showed correlation with pRNFL thickness whereas both MS-ON AEs and FEs did not. It is possible that the results observed in the MS-nON subgroup are indeed brought about by independent axonal loss or retro-chiasmal mechanisms, and those observed in the MS-ON subgroup have the additional element of demyelination caused by direct damage to the optic nerve, overwhelming the axonal effect.

### Test Stability

Finally, the tests were performed roughly 2 months apart in patients who have not undergone any therapeutic intervention or demyelinative episodes in the interim. The consistency of results obtained in these two assessments solidifies these measures as reliable tools for patient assessment in clinical and research setups. Additionally, the different information we gain by using the various measures tested emphasizes the importance of selecting the correct tool to answer the question studied, as we have recently discussed (32).

## Limitations and Future Research

This study was limited by the overall size of the group. In particular, the size of the subgroups used was limited, as some data could not be used in the MS-ON subgroup and due to the inclusion of patients with bilateral past ON in the encompassing study. This may have reduced the statistical power of the analysis. Another influence on statistical power may stem from the unequal sizes of the subgroups. An additional limitation which should be noted is the predominance of male participants in the study, despite the known female predominance in the disease itself. We have no explanation for this bias, however, the sex variable was included within the regression models and no statistical significance effect of sex was found.

Future research into this question should follow a cohort of MS patients with ON from early stages to later stages, attempting to capture the point in which the putative adaptive mechanisms fail under the onslaught of demyelination and disease load.

## Ethics Statement

This study was approved by the Hadassah-Hebrew University Medical Center Ethics Committee. All participants gave written informed consent.

## Author Contributions

YB designed and conceptualized the study, analyzed and interpreted the data, and drafted the manuscript. PP, HZ, RJ, MS, FP, and DK contributed to data analysis and revised the manuscript. HG-S and NR had a major role in data acquisition. NL designed and conceptualized the study and drafted the manuscript.

### Conflict of Interest Statement

The authors declare that the research was conducted in the absence of any commercial or financial relationships that could be construed as a potential conflict of interest.
