# Supplementary material for: Vision and Vision-Related Measures in Progressive Multiple Sclerosis
Source: Front Neurol. 2019 May 3;10:455. doi: 10.3389/fneur.2019.00455 (PMC6509148; doi:10.3389/fneur.2019.00455)
Supplement: Supplementary file 1 [file Table_1.DOCX]

Supplementary Material

Supplementary Table 1: Volume of Vision-Related Regions-of-Interest Per Group

| **Region-of-Interest** | **MS-ALL Volume (mm^3^)** | **MS-ON Volume (mm^3^)** | **MS-nON Volume (mm^3^)** |
| --- | --- | --- | --- |
| **Left SupParG** | 12.59937 | 12.72972 | 12.49799 |
| **Right SupParG** | 12.27685 | 12.46141 | 12.1333 |
| **Left SupMarG** | 8.551385 | 8.73211 | 8.410822 |
| **Right SupMarG** | 8.094556 | 8.232629 | 7.987167 |
| **Left AngG** | 10.49409 | 10.69622 | 10.33688 |
| **Right AngG** | 11.81101 | 12.03525 | 11.6366 |
| **Left PCu** | 6.814069 | 6.765529 | 6.851822 |
| **Right PCu** | 6.887031 | 6.923576 | 6.858607 |
| **Left SupOccG** | 3.97631 | 4.060414 | 3.910896 |
| **Right SupOccG** | 4.474408 | 4.617252 | 4.363307 |
| **Left MidOccG** | 11.85198 | 12.01179 | 11.72768 |
| **Right MidOccG** | 12.1293 | 12.3655 | 11.94559 |
| **Left InfOccG** | 6.202821 | 6.289781 | 6.135185 |
| **Right InfOccG** | 6.408006 | 6.52209 | 6.319274 |
| **Left Cun** | 3.420992 | 3.551871 | 3.319196 |
| **Right Cun** | 3.963985 | 4.075405 | 3.877326 |
| **Left InfTemG** | 16.5012 | 16.79624 | 16.27173 |
| **Right InfTemG** | 15.44811 | 15.57684 | 15.34799 |
| **Left ParHipG** | 14.9584 | 15.19769 | 14.77229 |
| **Right ParHipG** | 14.78091 | 15.16904 | 14.47903 |
| **Left LinG** | 12.81949 | 12.96772 | 12.70421 |
| **Right LinG** | 13.72152 | 13.90963 | 13.5752 |
| **Left FusG** | 4.079294 | 4.220162 | 3.96973 |
| **Right FusG** | 4.296692 | 4.382167 | 4.230211 |

SupParG = Superior Parietal Gyrus; SupMarG = Supramarginal Gyrus; AngG = Angular Gyrus; PCu = Precuneus; SupOccG = Superior Occipital Gyrus; MidOccG = Middle Occipital Gyrus; InfOccG = Inferior Occipital Gyrus; Cun = Cuneus; InfTemG = Inferior Temporal Gyrus; ParHipG = Parahippocampal Gyrus; LinG = Lingual Gyrus; FusG = Fusiform Gyrus.
